# Comparative Evaluation of Different Methods of Activation of Chelating Solution for Smear Layer Removal in the Apical Portion of the Root Canal Using a Scanning Electron Microscopy: An In Vitro Study

**DOI:** 10.7759/cureus.66622

**Published:** 2024-08-11

**Authors:** Mrunal B Alhat, Sudha B Mattigatti, Rushikesh R Mahaparale, Kapil D Wahane, Apoorva Jadhav

**Affiliations:** 1 Department of Conservative Dentistry and Endodontics, Krishna Vishwa Vidyapeeth (Deemed to be University), Karad, IND

**Keywords:** chelating agents, root canal irrigants, endoactivator, root canal brush, xp-endo finisher, smear layer

## Abstract

Background

The smear layer has an adverse effect on the sealing of root canals during obturation, and it is the main reason for the failure of root canal treatment. Root canal irrigation using a conventional irrigation system is ineffective for smear layer removal, especially from the apical third region, where most lateral canals are present. For successful endodontic treatment, the smear layer should be removed from the apical third region using an effective irrigation activation system.

Aim

This study aimed to compare smear layer removal by conventional irrigation, XP Endo Finisher (XPF), endoactivator (EA), passive ultrasonic activation, and root canal brush using 17% ethylenediaminetetraacetic acid (EDTA) as a chelating solution and 5.25% sodium hypochlorite after chemomechanical preparation, using scanning electron microscopy (SEM).

Method

A total of 50 extracted human mandibular single canal premolars with mature roots were selected for this study. Samples were decoronated to obtain a standard working length (WL) of 15 mm. Canal patency was achieved using a 10 k file. Samples were sealed with sticky wax to obtain the vapor lock effect. Biomechanical preparation is done till F3. The samples were divided into five groups according to the final irrigation activation protocol: Group 1, control group; group 2, XPF; group 3, EA; group 4, passive ultrasonic irrigation (PUI); and group 5,, root canal brush. Samples were divided into two equal halves longitudinally. Each sample was analyzed for a smear layer under SEM at 2000x magnification. Statistical analysis was done using the one-way Anova "F" test and Tukey’s post-hoc test.

Results

Group 3 showed the least presence of a smear layer, followed by groups 4, 2, 5, and 1. All the groups exhibited highly significant differences between each other (p < 0.001). Group B shows no significant difference with groups C, D, and E. Group C shows no significant difference with groups D and E. Group D shows no significant difference with group E.

Conclusion

The EA removes the smear layer effectively as compared with other groups. All the irrigation activation system shows the presence of smear layer. No activation systems were able to remove the smear layer completely.

## Introduction

Endodontic therapy includes eliminating bacteria and infected dentin, which involves straight-line access to root canals; complete debridement with meticulous biomechanical preparation; disinfection; and lastly, complete obturation [1]. Cleaning and shaping have a direct impact on the filling and disinfection of canals; therefore, the main objective of endodontic treatment is to eliminate every microbe found inside the root canal.

Apical leakage is a more repeated cause of endodontic treatment failure [2]. Organic pulp remnants and inorganic dentinal debris build over the canal surface during the use of manual and rotary instruments. The development of a sludge layer known as the "smear layer" across the surfaces of instrumented root canal walls was documented by McComb and Smith [3]. Dentine shavings, cell debris, and pulp remnants make up this smear layer, which is divided into two different strata: an attached stratum that produces occluding plugs inside dentinal tubules and a loose surface deposit [4]. The smear contains necrotic pulps, microorganisms, as well as their byproducts. The smear layer's particles have sizes between >0.5 and 15 µm, while the smear layer's thickness varies between 1 and 5 µm.

Independent of the endodontic filling method, various authors reported that canal surfaces lacking smears allow filling materials to enter patent dentinal tubules, increasing the contact surface, enhancing mechanical retention, and lowering the likelihood of microleakage through the filled canal. It is important to eliminate these extraneous layers of both organic and inorganic debris prior to the root canal being sealed [5]. According to Haapasalo et al., removal of the smear may make it easier for intracanal medications to enter dentinal tubules of infected root canals, improving the disinfection process [5].

On the other aspect, other researchers think that while the canal is being prepared, the smear should be kept because by changing permeability, it can prevent dentinal tubules from exchanging germs and other irritants [6]. According to Williams and Goldman, the smear layer could only delay bacterial entry and does not act as a barrier [7]. Nevertheless, there is significant evidence that the smear layer should be removed [8]. A total of 93% of lateral and auxiliary canals in the apical area of the root canal have reported a smear layer. Failure of endodontic treatment and apical microleakage occur because of the existence of a smear layer in the apical third area. A clean, smear-layer-free apical third area is necessary for successful endodontic therapy.

There are several activation procedures and solutions available that are used to remove smear layers. Ultrasound in endodontics was studied in the 1950s. Prati et al. achieved the eradication of the smear layer with ultrasonics [9]. Passive ultrasonic irrigation (PUI) is therefore employed to remove the smear layer. The conventional method of irrigation with a needle syringe that moves up and down is inefficient for removing smear layers. A microbrush attached to a rotating handpiece is used as a rotary root canal brush. A tapered brush part and a shaft on the brush are present. The central core of the wire supports several bristles that extend in all directions [10]. The frequency of ultrasonic irrigation is greater (25-30 kHz), which results in increased shear stress. The principles of piezoelectric and acoustic microstreaming are the basis for ultrasonic irrigation. Sonic irrigation operates at a lower frequency and with less shear force (1-6 KHz). Tronstad et al. discovered that the endoactivator (EA), a kind of sonic irrigation used for treatment, uses noncutting polymer tips and works on hydrodynamic phenomena [11]. A unique kind of file system called an XP Endo Finisher (XPF) is utilized to activate irrigation. A unique proprietary alloy of nickel-titanium (NiTi) is used to make XPF [12].

The aim of this research study is to compare smear layer removal by conventional irrigation, XPF, passive ultrasonic activation, root canal brushing, and EA using 17% ethylenediaminetetraacetic acid (EDTA) as a chelating solution 5.25% sodium hypochlorite after chemomechanical preparation, using scanning electron microscopy (SEM).

## Materials and methods

A power analysis was obtained using G*Power version 3.0.1 (Franz Faul Universitat, Kiel, Germany). The total minimum calculated size of samples are 50, i.e., 10 samples per group, which gives 80% power for showing significant differences, having an effect size of 0.52 and also a significance level of 0.05. Fifty permanent single-rooted teeth and lower premolars were selected for this in vitro study. The teeth have been obtained from patients of all ages and genders. The teeth were extracted for orthodontic treatment of periodontal diseases and excluded with grossly decayed or carious, fractured, teeth with curved roots. A diamond disk was utilized for sectioning the teeth at the cementoenamel junction (CEJ). The working length (WL) was kept at 15 mm. 

**Figure 1:**
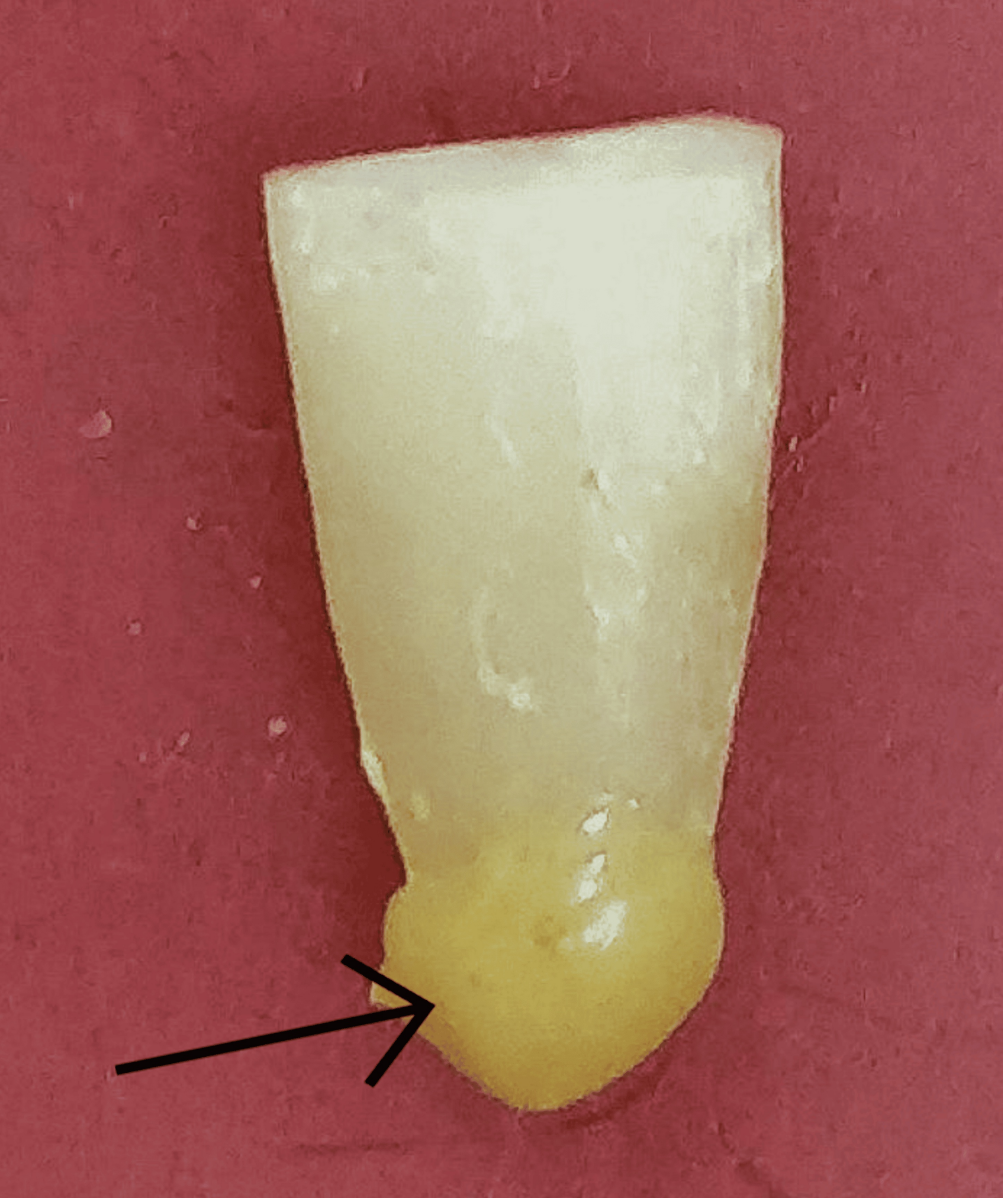
Sealed with a sticky wax

Prior to canal preparation, the patency of the root canal was verified by passing a #10 hand K-file (Mani, Japan) through the apical foramen. A sticky wax was used to mimic the vapor lock effect on 2 mm of root apices (Figure 1).

**Figure 2:**
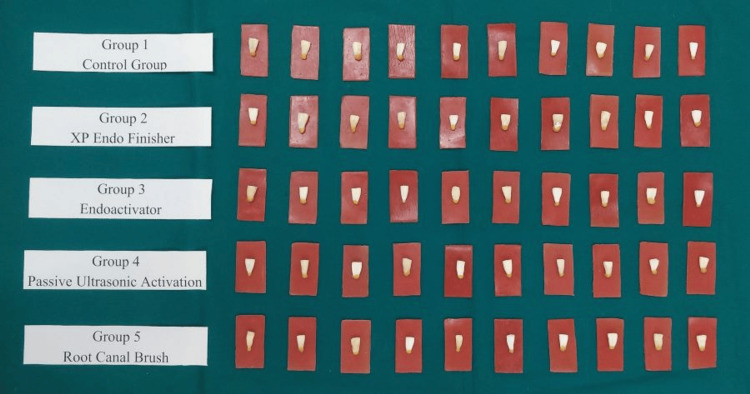
Samples divided into five groups

Using ProTaper rotary files (Dentsply ProTaper Universal Rotary, Germany) up to F3, every root canal was prepared in a crown-down manner. Canals were recapitulated with #20 K-file (Mani, Japan) and irrigated with 2 mL of 5.25% NaOCl (Prime Dental, India). Following that, absorbent paper points were utilized for drying the canals, and cotton pellets were placed there to cover the canal orifices. The teeth were separated arbitrarily within five groups (n = 10) (Figure 2). The final irrigation procedures were performed. The irrigation techniques were used.

Group A: control group

**Figure 3:**
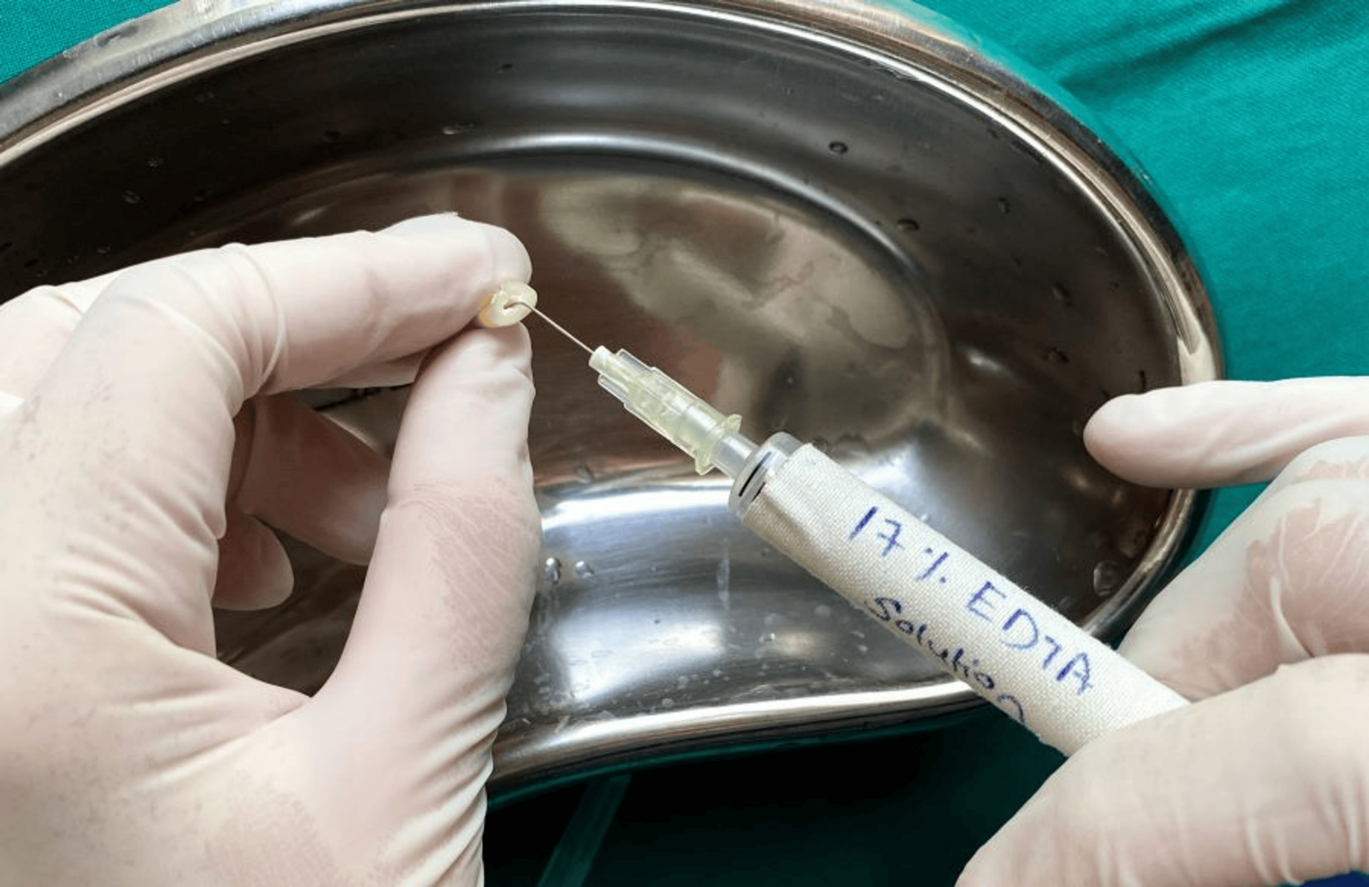
Irrigation using a side vented needle

The 30-gauge NaviTip double-sideport needle (Waldent, China) was calibrated to reach 1 mm short from the WL, and 2.5 mL of 17% EDTA solution (Ammdent Canalarge, India) was administered to irrigate the canals. No more irrigant activation was carried out (Figure 3).

Group B: XPF (FKG Swiss Endo, Switzerland)

**Figure 4:**
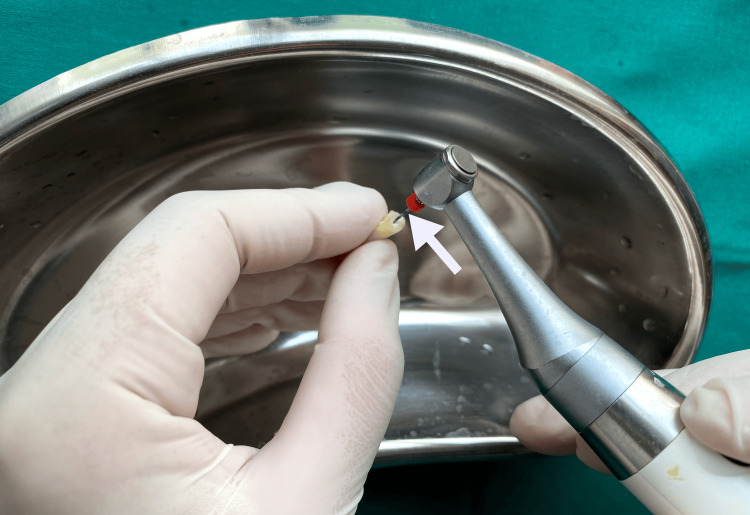
Irrigation activation using XP Endo Finisher

The 30-gauge NaviTip double-sided needle was calibrated to reach 1 mm short of the WL, and 2.5 mL of 17% EDTA solution was administered to irrigate the root canals. The XPF file was used to activate the irrigating agent for one minute, revolving at 800 rpm and achieving the WL. The XPF file was moved slowly and gently in a 7-8 mm longitudinal direction (Figure 4).

Group C: EA (Dentsply, Germany)

**Figure 5:**
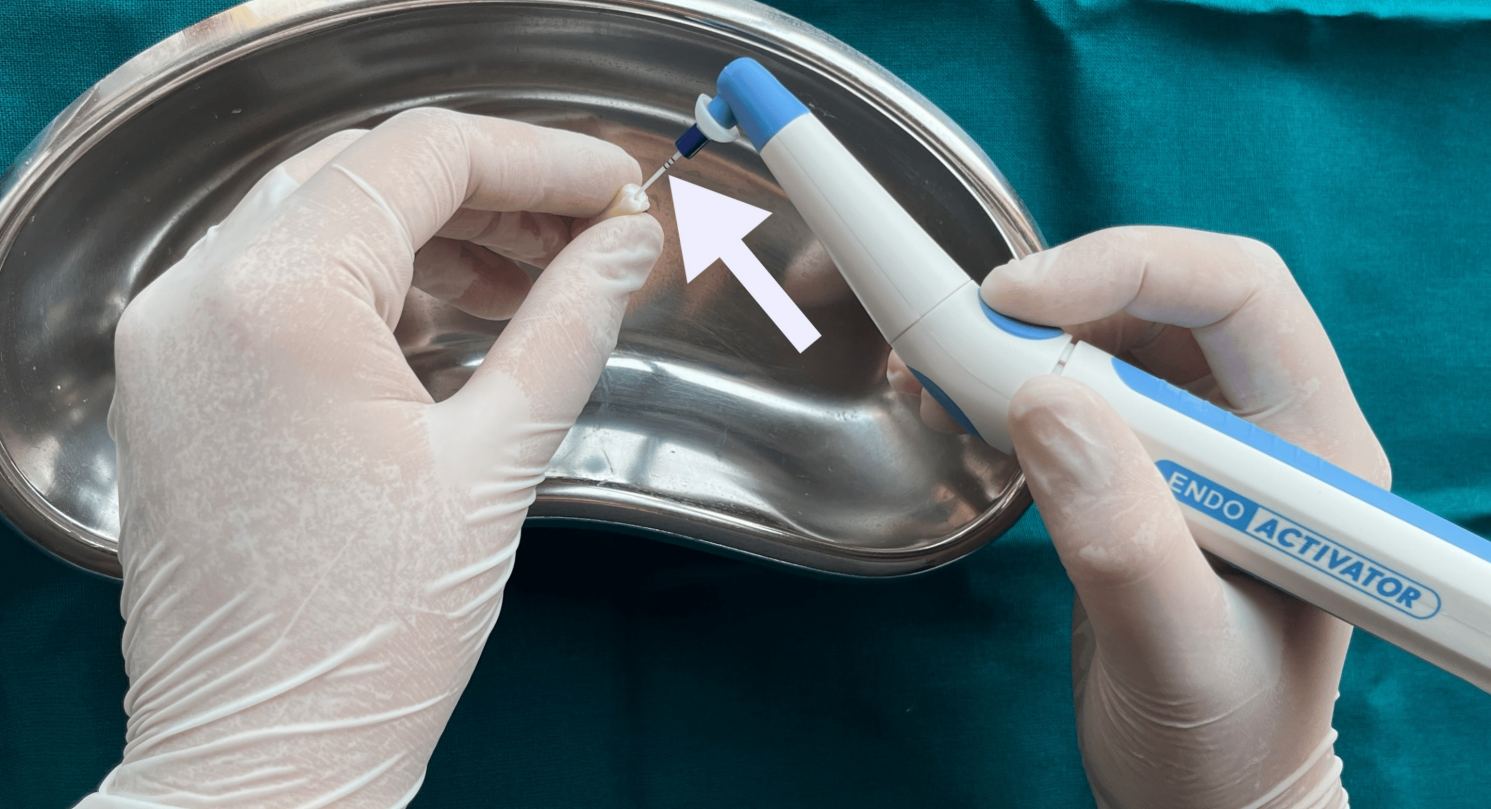
Irrigation activation using an endoactivator

The 30-gauge NaviTip double-sided needle was calibrated to reach 1 mm short of the WL, and 2.5 mL of 17% EDTA solution was administered to irrigate the root canals. Activated for one minute using the EA blue tip at 10,000 cycles per min (Figure 5).

Group D: passive ultrasonic activation (Woodpecker, China)

**Figure 6:**
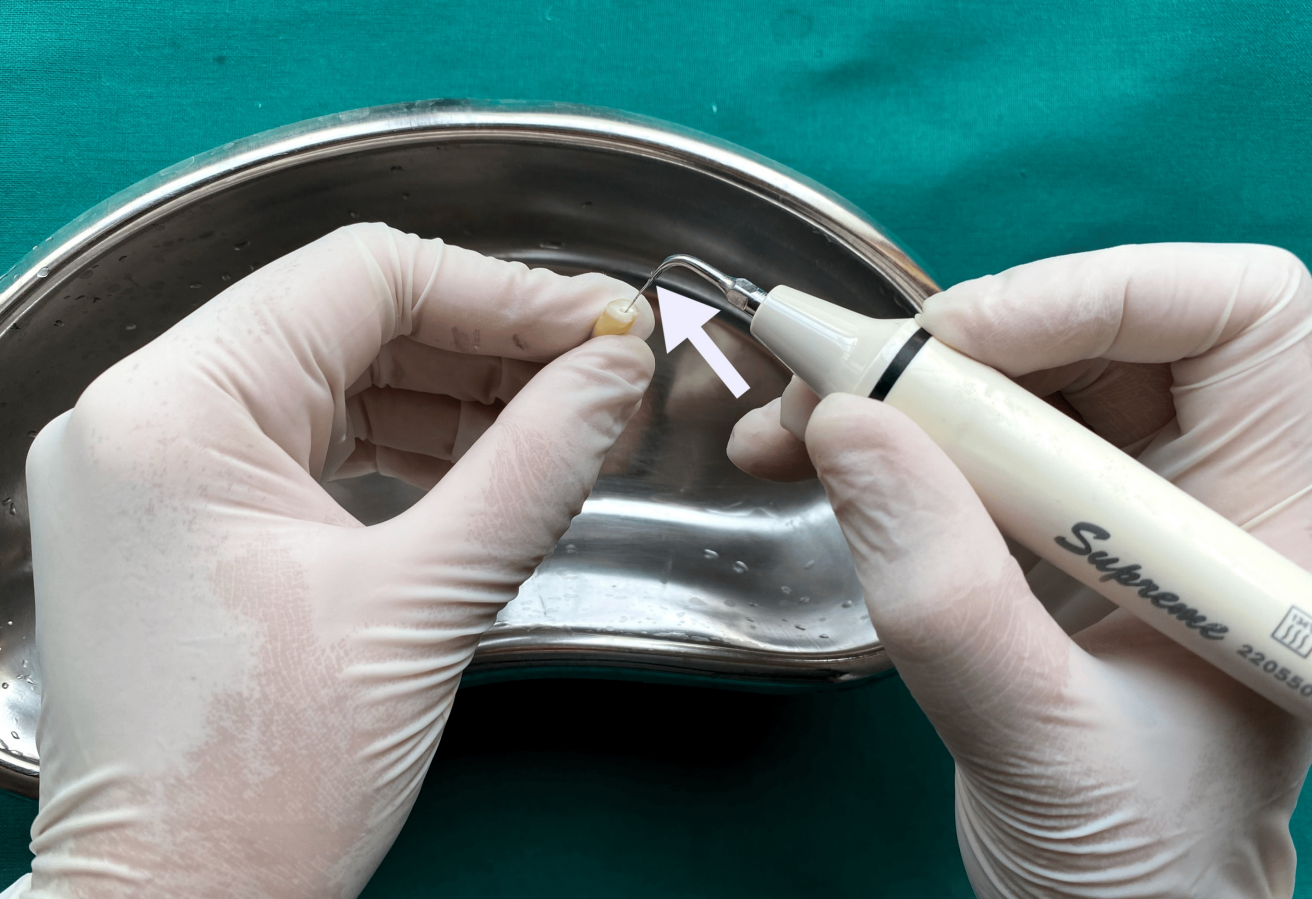
Irrigation activation using a passive ultrasonic irrigation tip

The 30-gauge NaviTip double-sided needle was calibrated to reach 1 mm short of the WL, and 2.5 mL of 17% EDTA solution was administered to irrigate the root canals. Activation was done for one minute using an ultrasonic tip at power setting 4. Tip is placed 2 mm short of the WL (Figure 6).

Group E: root canal brush (Coltene, India)

**Figure 7:**
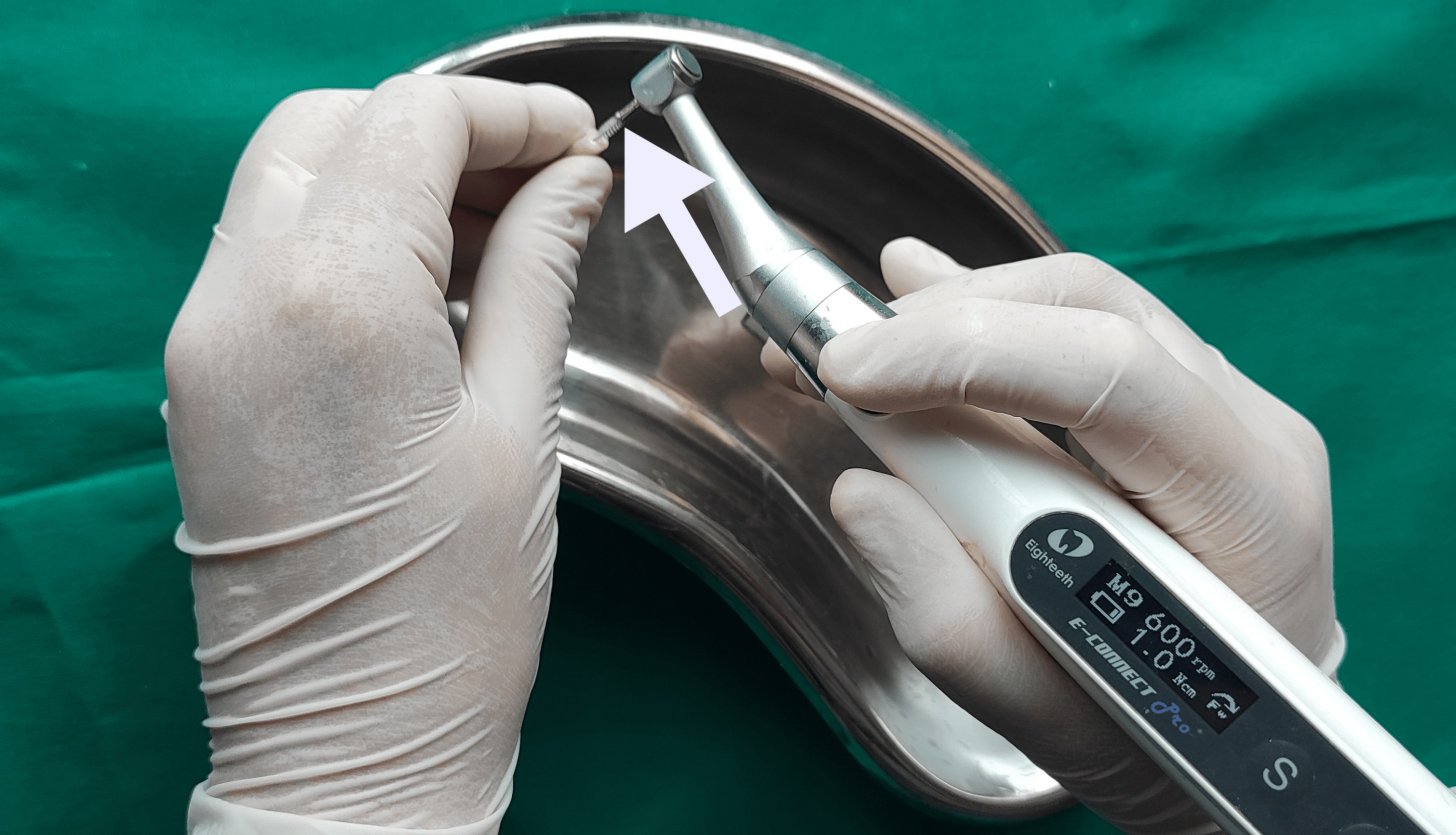
Irrigation activation using root canal brushes

The 30-gauge NaviTip double-sided needle was calibrated to reach 1 mm short of the WL, and 2.5 mL of 17% EDTA solution was administered to irrigate the root canals. Activated for one minute with a contrangle handpiece set at 600 rpm and a polypropylene CanalBrush (Figure 7).

**Figure 8:**
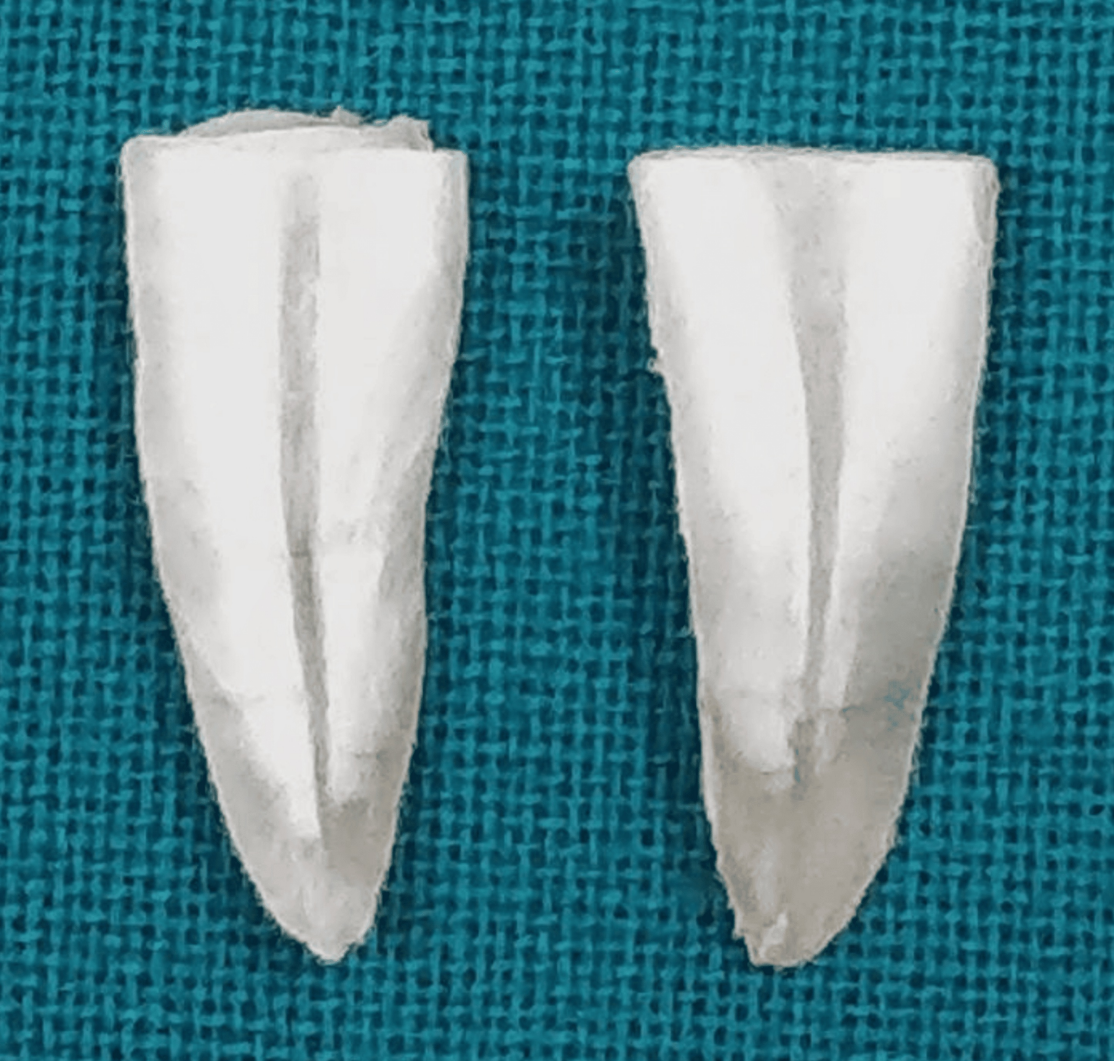
Sections of a single tooth

**Figure 9:**
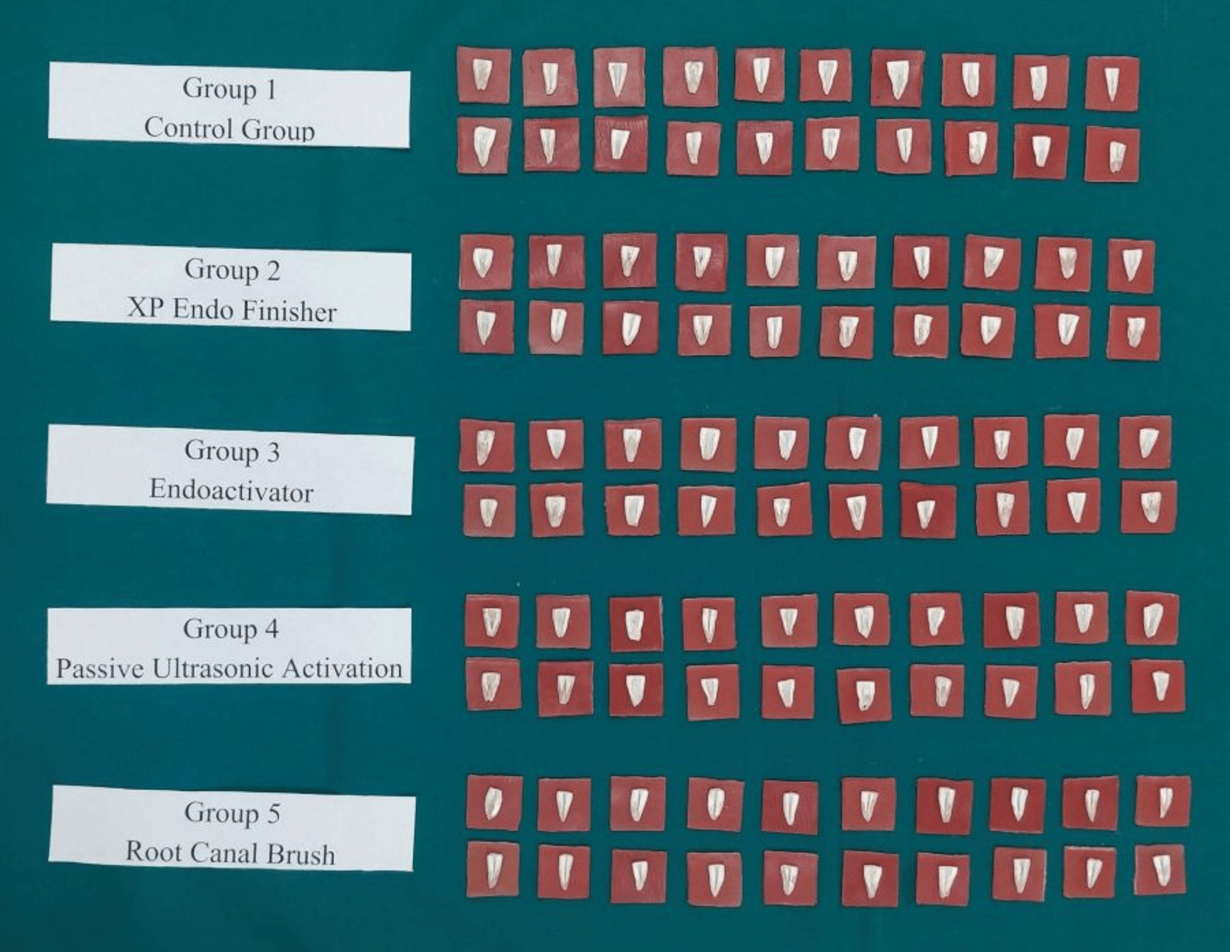
Sections of all samples

After that, canals were irrigated with 2.5 mL of 5.25% NaOCl, aspirated, and dried with absorbent paper points. A longitudinal groove was created in the buccolingual direction using a diamond disk. In order to prevent the cutting disk from encroaching into the canals, colored gutta-percha cones were inserted in the canals and utilized as markers. Two separated portions were created from every sample by applying gentle pressure to the roots with a chisel in the vertical groove (Figures 8-9).

**Figure 10:**
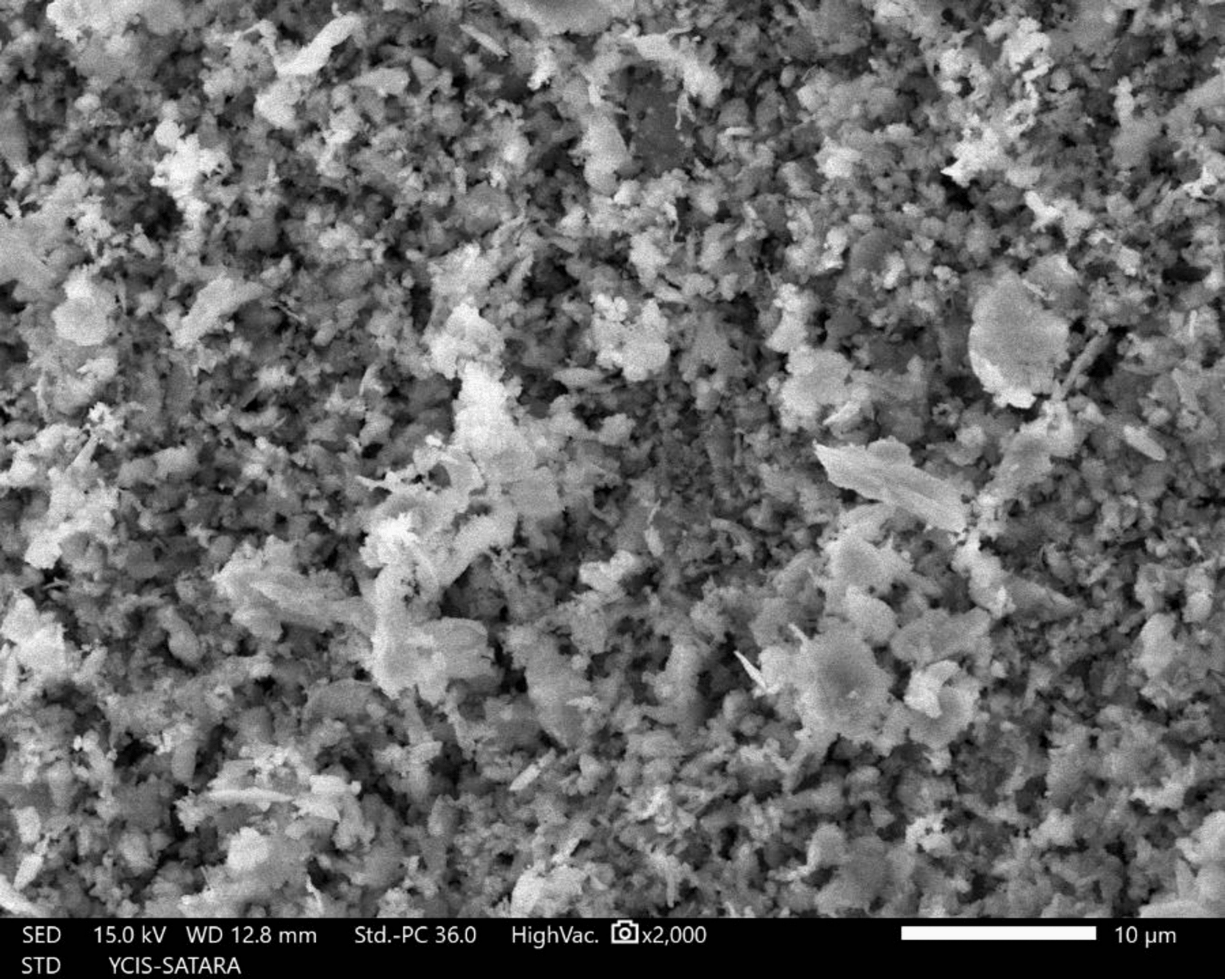
SEM image of group 1 SEM: Scanning electron microscopy

**Figure 11:**
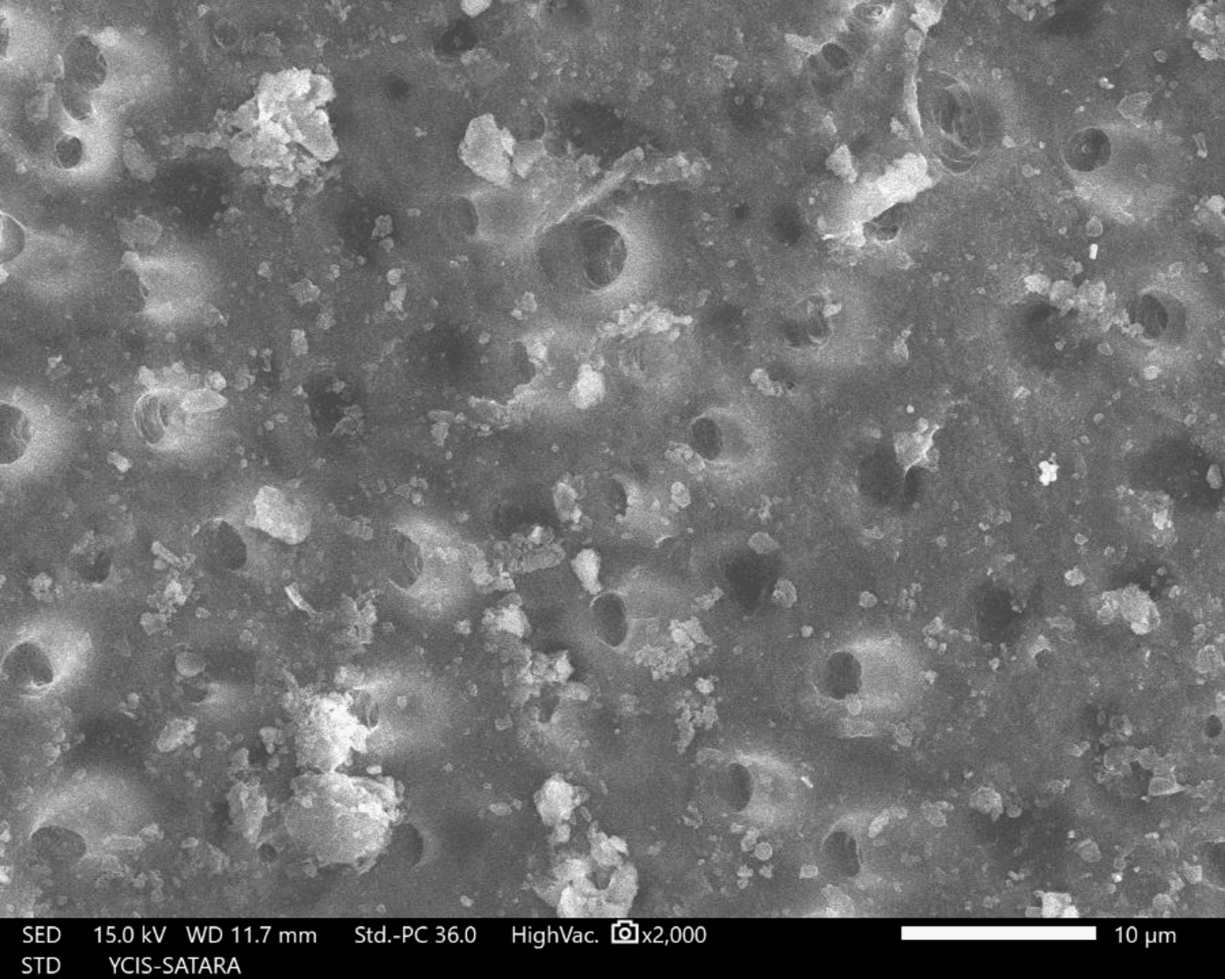
SEM image of group 2 SEM: Scanning electron microscopy

**Figure 12:**
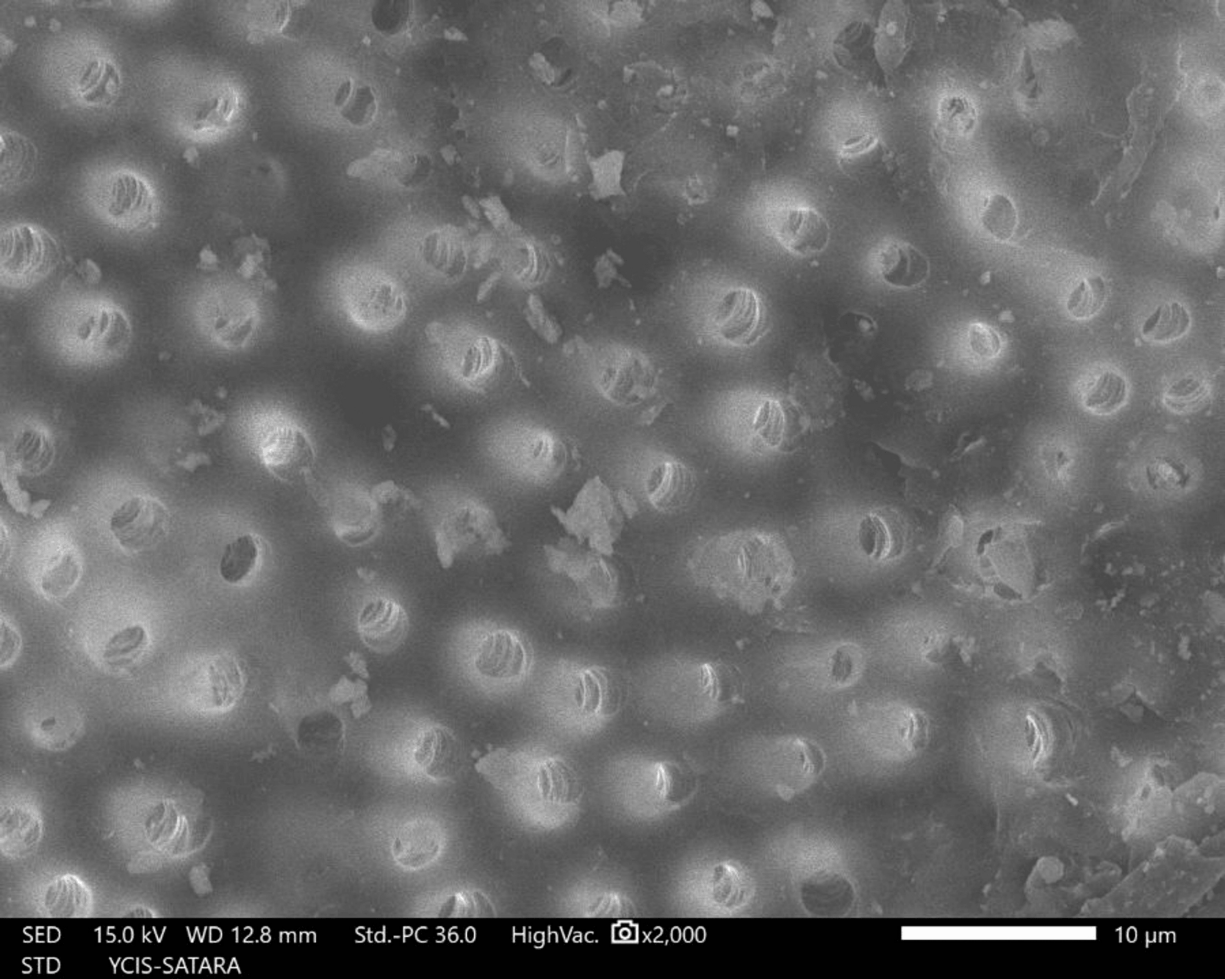
SEM image of group 3 SEM: Scanning electron microscopy

**Figure 13:**
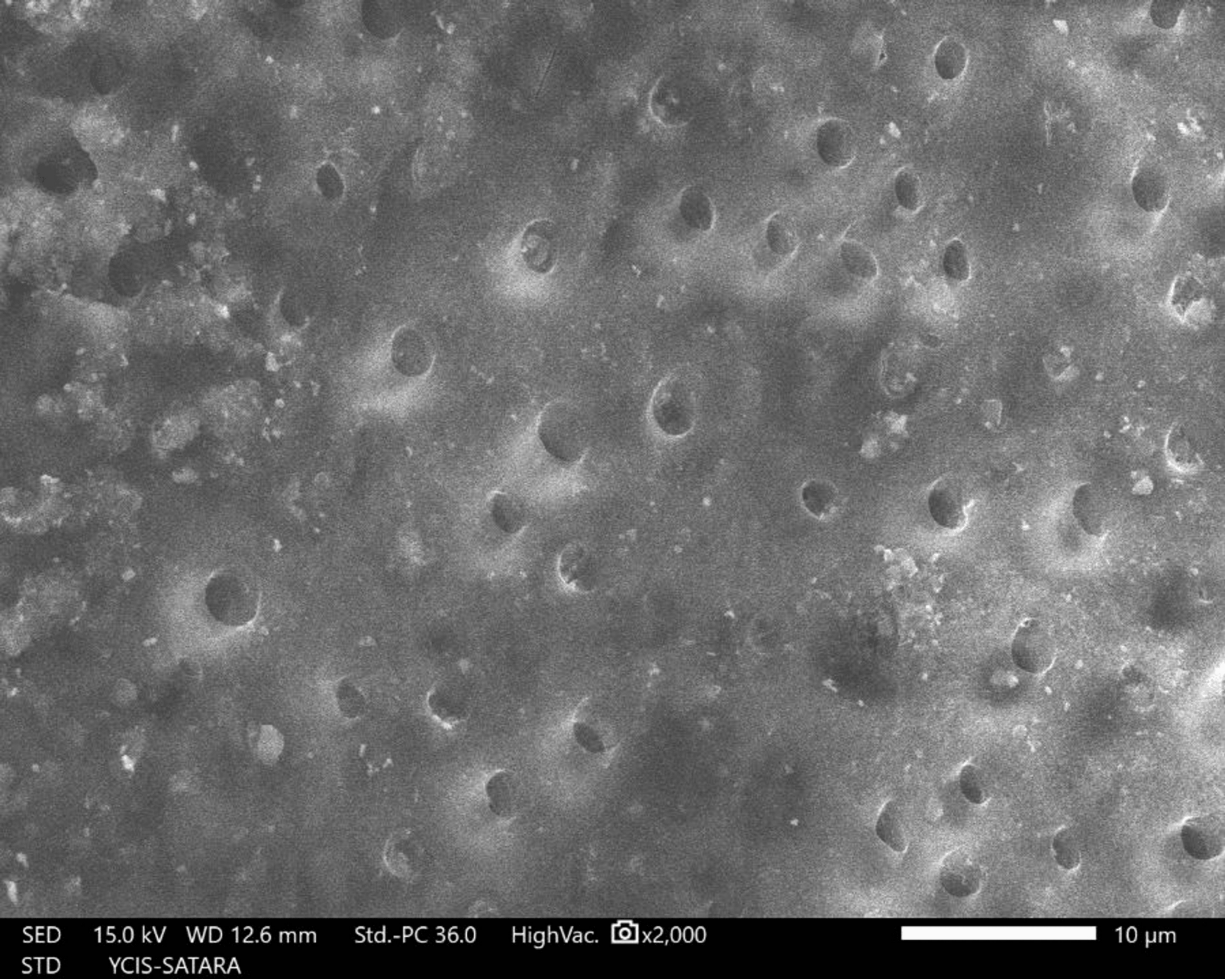
SEM image of group 4 SEM: Scanning electron microscopy

**Figure 14:**
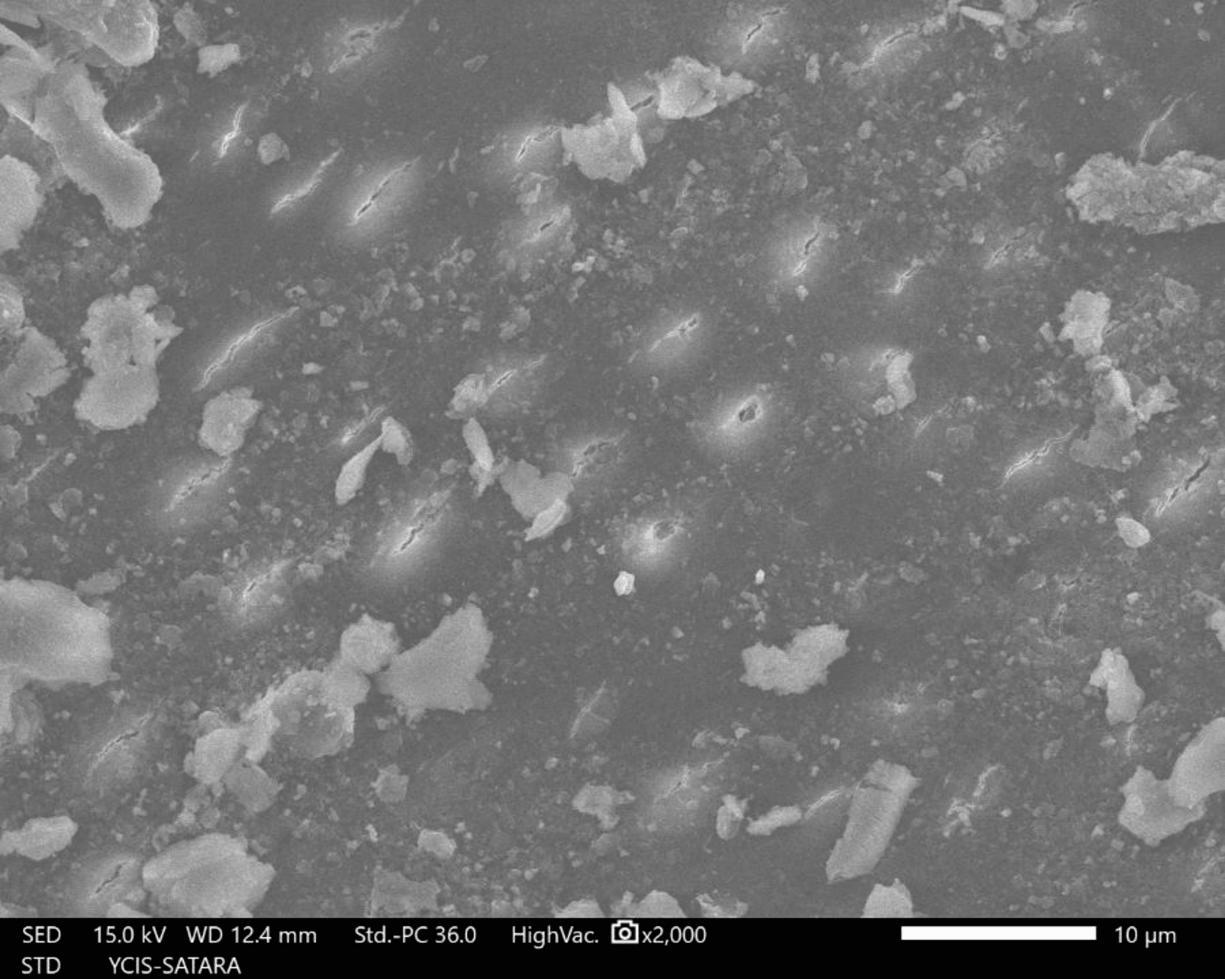
SEM image of group 5 SEM: Scanning electron microscopy

The sections were dehydrated for eight hours using 50%, 70%, 90%, and 100% ethyl alcohol. Followed by air-drying in a desiccator for 72 hours, sputter-coated with a 30 nm layer of gold and palladium, mounted on aluminum stubs, and examined with SEM. Using a SEM, images of the apical third area for each hemisection were taken at a magnification of ×2,000. SEM images of group 1 (Figure 10), group 2 (Figure 11), group 3 (Figure 12), group 4 (Figure 13), and group 5 (Figure 14).

Method of evaluating the smear layer

**Figure 15:**
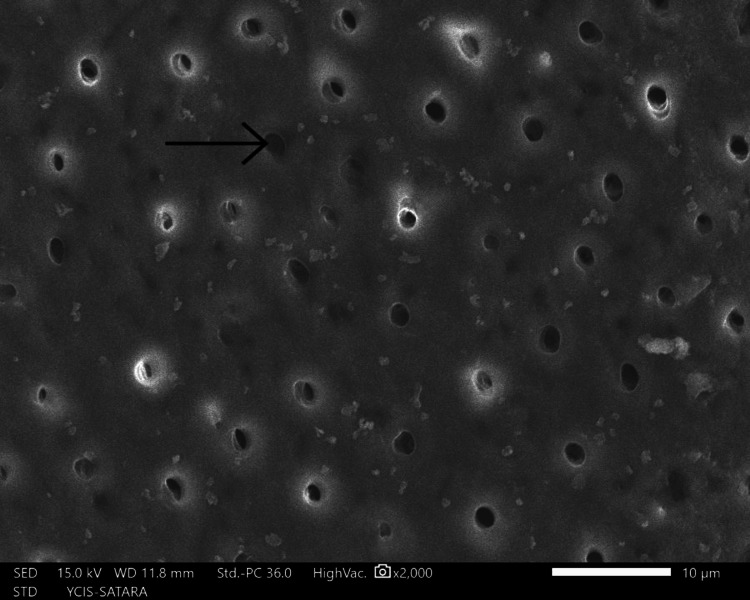
Score 1

**Figure 16:**
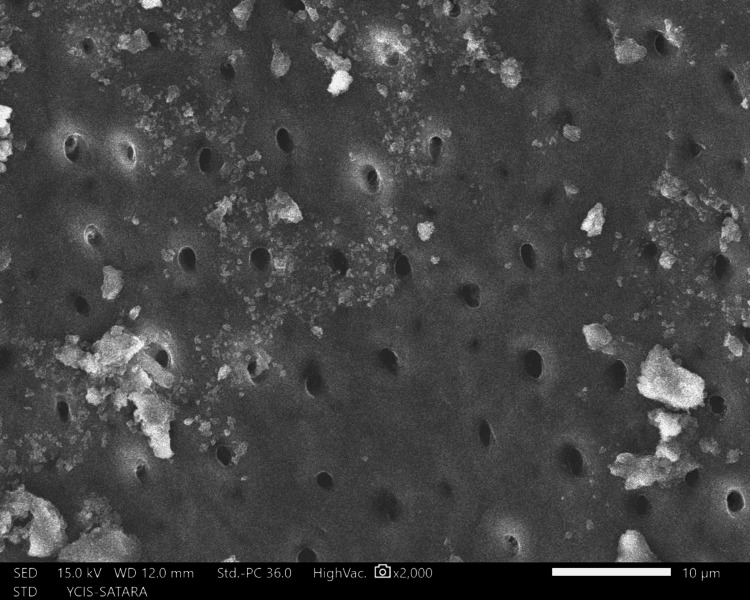
Score 2

**Figure 17:**
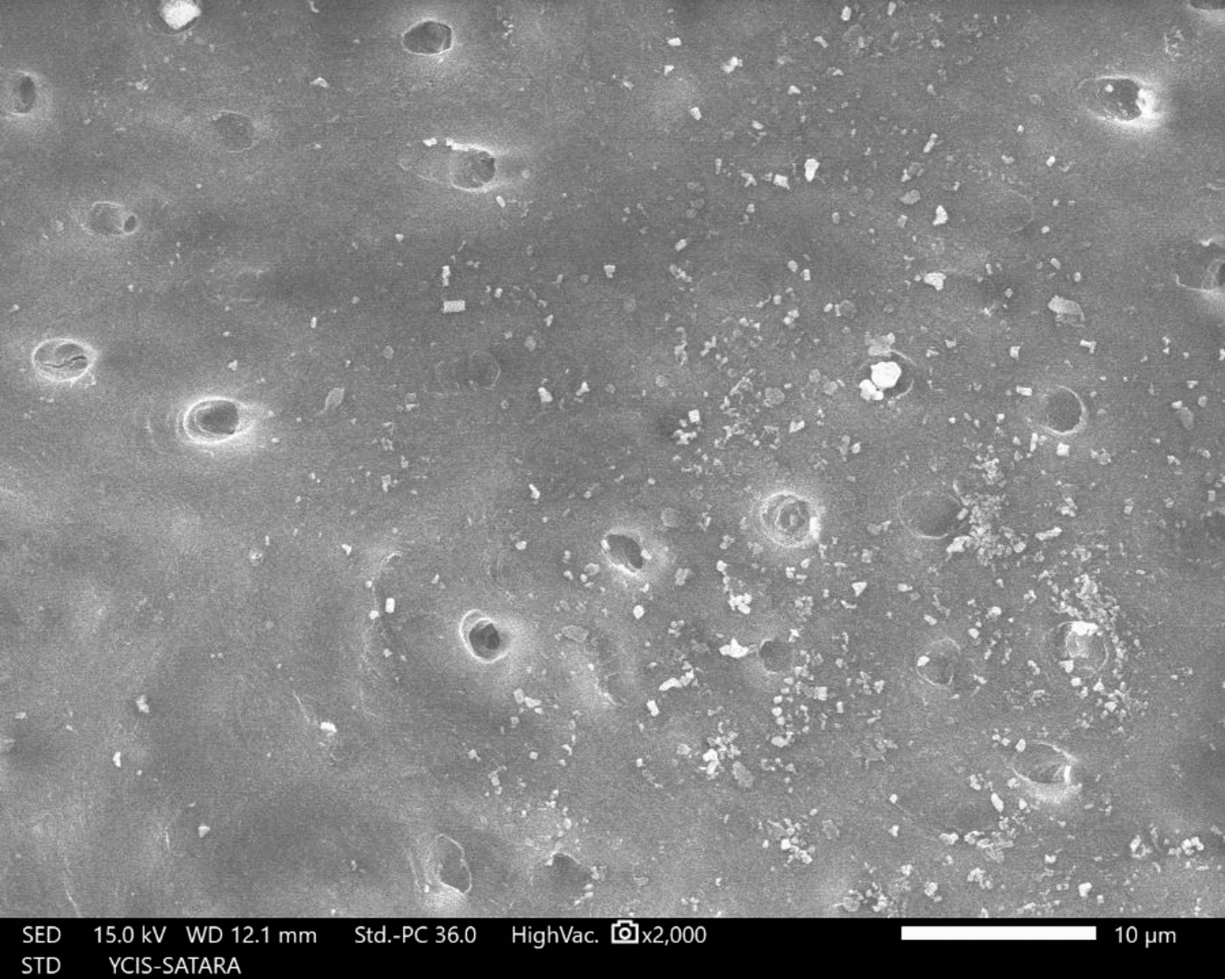
Score 3

**Figure 18:**
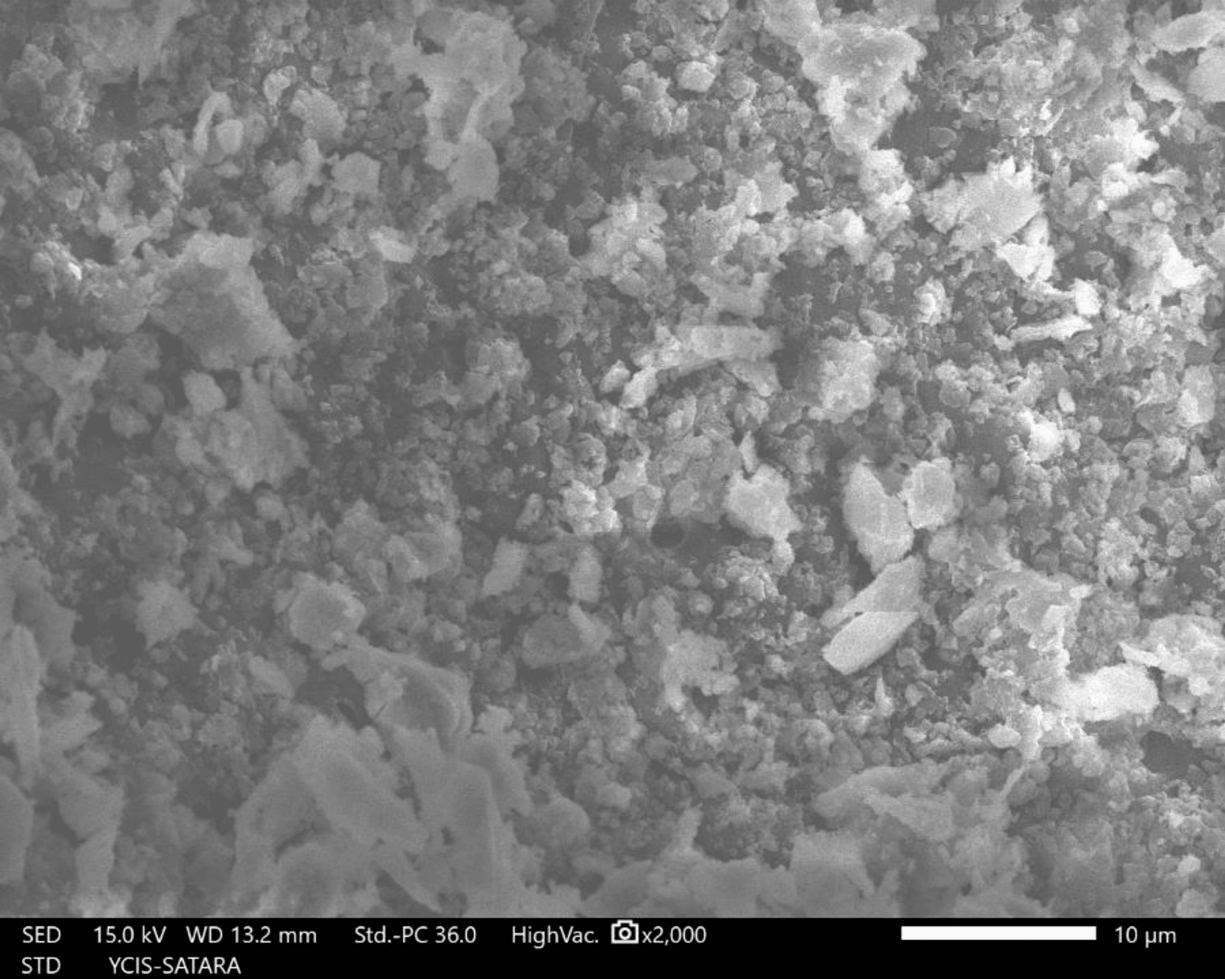
Score 4

**Figure 19:**
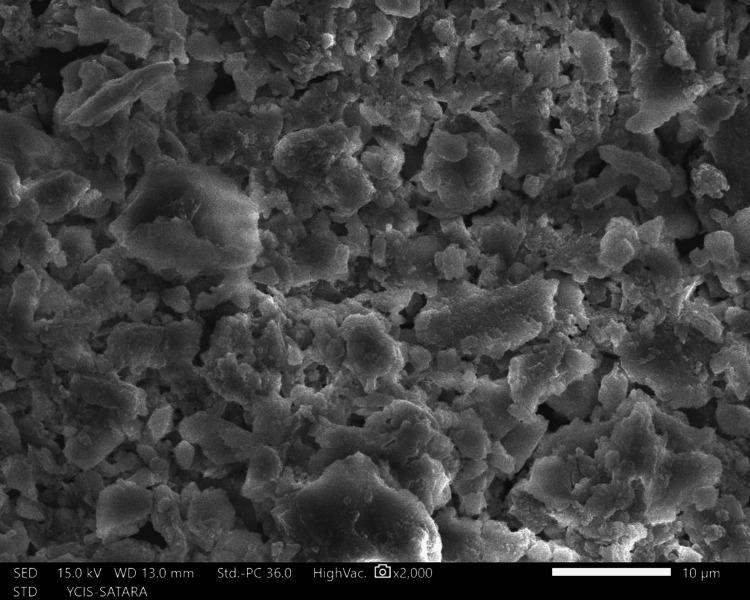
Score 5

The following ratings were utilized for the analysis of the smear surface: score 1 = absence of smear layer, patent dentinal tubule orifices (Figure 15); score 2 = a small quantity of opened dentinal tubules, presence of smear (Figure 16); score 3 = nearly the complete canal walls have a uniform layer of smear, with very few opened tubules of dentine (Figure 17); score 4 = the whole canal wall with the absence of opened tubules of dentine, coated in a uniform smear layer (Figure 18); and score 5 = a thick layer of uniform smears that completely covered the canal wall (Figure 19) [13].

Statistical analysis

IBM SPSS Statistics for Windows, Version 21 (Released 2012; IBM Corp., Armonk, New York, United States) was utilized to conduct the statistical analysis. The Shapiro-Wilk test was utilized to verify data normality. The alpha error (the level of significance) is set at 5%, and the confidence interval is set at 95%. The overall group comparison was done using a parametric test, namely, the one-way analysis of variance (ANOVA) F test. Afterward, pairwise intergroup comparison was done using Tukey's post hoc test. A p-value of less than or equal to 0.05 (p < 0.05) was considered statistically significant in the tests mentioned above.

## Results

The mean values for the presence of the smear layer show the least value for group C followed by groups D, B, E, and A (Table 1).

**Table 1:** Descriptive statistics of smear layer removal SD: Standard deviation; SE: standard error

	Mean	SD	SE	Minimum	Maximum
Group A (control group)	4.3	0.73	0.16	3.0	5.0
Group B (XP Endo Finisher)	2.55	0.51	0.11	2.0	3.0
Group C (endoactivator)	2.4	0.68	0.15	1.0	3.0
Group D (passive ultrasonic)	2.45	0.51	0.11	2.0	3.0
Group E (root canal brush)	2.8	0.41	0.09	2.0	3.0

For overall intergroup comparison using the one-way ANOVA F test shows highly significant differences between each other (p < 0.001) (Table 2).

**Table 2:** Overall intergroup comparison for smear layer removal by using one-way ANOVA F test SD: Standard deviation; p < 0.001: highly statistical significant difference

	Mean	SD	One-way ANOVA F test	p-value, significance
Group A (control group)	4.3	0.73	F = 37.66	p < 0.001**
Group B (XP Endo Finisher)	2.55	0.51		
Group C (endoactivator)	2.4	0.68		
Group D (passive ultrasonic)	2.45	0.51		
Group E (root canal brush)	2.8	0.41		

Pairwise group comparison using Tukey's post hoc test shows highly significant difference present in between group A and group B, group A and group C, group A and group D, and group A and group E. No significant difference was present in between group B and group C, group B and group D, group B and group E, group C and group D, group C and group E, and group D and group E (Table 3).

**Table 3:** Pairwise intergroup comparison for smear layer removal by using Tukey’s post hoc test p > 0.05: No significant difference (NS); *p < 0.05: significant; **p < 0.001: highly significant

Tukey’s post hoc test for pairwise comparison			
Group	Comparison group	Mean difference	p-value, significance
Group A (control group) vs	Group B (XP Endo Finisher)	1.75	p < 0.001**
	Group C (endoactivator)	1.9	p < 0.001**
	Group D (passive ultrasonic)	1.85	p < 0.001**
	Group E (root canal brush)	1.5	p < 0.001**
Group B (XP Endo Finisher) vs	Group C (endoactivator)	0.15	p = 0.925 (NS)
	Group D (passive ultrasonic)	0.1	p = 0.982 (NS)
	Group E (root canal brush)	0.25	p = 0.655 (NS)
Group C (endoactivator) vs	Group D (passive ultrasonic)	0.05	p = 0.999 (NS)
	Group E (root canal brush)	0.40	p = 0.198 (NS)
Group D (passive ultrasonic) vs	Group E (root canal brush)	0.35	p = 0.322 (NS)

## Discussion

Standard irrigation procedure involves syringe irrigation, yet this method is ineffective over the apical region of the root canal. The apical one-third region of the root is smaller when differentiated from the other thirds, making it very difficult to fully eliminate the leftover smear layer since it obstructs the irrigating solutions' ability to circulate and act. Additionally, it was demonstrated that smears prevent intracanal disinfectants and sealants from penetrating dentinal tubules and may jeopardize the root filling's seal. Various techniques have been introduced to get rid of it [14]. Compared to manual irrigation using a syringe and needle, further activation of the irrigants may improve the removal of debris and smear from the canal system.

Single-rooted premolar teeth with a single canal were utilized for this research study. This was carried out to standardize research. To provide more consistency in the research sample, the lengths of the teeth were standardized in the sample under investigation. To do this, the crowns were also cut to provide a stable reference point for the working length [15]. To make sure the canal was prepared to the required standard width, the biomechanical preparation was completed up to F3 ProTaper. To replicate the vapor-lock phenomenon, a sticky wax was applied to the apex of the samples. Since the root is encased by the bone socket in this instance, the canal functions as a closed-end space. As a result, in vivo cleaning and shaping may cause gas to become trapped inside the root canal, which results in vapor-lock effect formation [16].

The most used technique for the evaluation of smear layer removal is SEM. To lessen the detrimental effects of subjectivity, the study's photos were taken at magnifications of ×2,000, which made it possible to analyze each specimen over the apical region in more detail [17].

Sodium hypochlorite is a widely utilized irrigant in endodontics because of its antibacterial qualities and ability to dissolve tissue. Urban et al. suggest that in order to remove the smear layer effectively, a chelating solution must be used. EDTA has shown higher performance in eradicating the smear layer during the final irrigation [18]. The inorganic part of the smear is eradicated by EDTA, while the organic portion is removed by sodium hypochlorite.

A syringe and needle are used in the usual irrigation technique during endodontic therapy. Because consistent irrigant delivery up to the WL using needle irrigation may not be achieved, it could be useful for cleaning the coronal one-third area of root canals but ineffective for cleaning the apical third. This is because of the irrigant's limited ability to go more than 1 mm over the needle's tip. Vapor lock stops the irrigant from efficiently reaching the WL and gas entrapment at its closed end [19].

Results of this research showed that the EA (2.4 ± 0.68) shows less smear in the apical third region after that PUI (2.45 ± 0.51), XPF (2.55 ± 0.51), root canal brushes (2.8 ± 0.41), and control group (4.3 ± 0.73).

The EA uses a hydrodynamic phenomenon to eliminate the smear. The EA tips that are 22 mm in length, flexible, and color-coded according to size (yellow: 15/02; red: 25/04; blue: 35/04). To enhance the final irrigation technique, it may be employed at three different speeds: 10,000; 6,000; and 2,000 cycles per minute. This will provide a 160/190 Hz frequency [20].

Activating passive ultrasonic systems is among the most well-liked and often utilized irrigation systems. By using cavitation and acoustic microstreaming, PUI might enhance cleaning effects in intricate canal anatomic locations. The method involves the passive insertion of a metal tip or file into a canal that has a chelating or irrigating solution inside of it. The tip or file is connected to an ultrasonic instrument that oscillates at a frequency of 30 KHz [21].

The results of this research are corroborated by Caron et al., who demonstrated that the EA considerably improved smear layer clearance compared to no agitation [22]. Kharod et al. and Khalap et al. conducted a number of tests and came to the conclusion that the EA's sonic agitation of irrigant was more impactful than PUI systems in getting rid of debris and smear layers. They claimed that the reason for this outcome was the unintended dampening impact of the amplitude of its distinctive nodes and antinodes pattern, particularly when the instrument met the lateral walls of a curved canal during PUI [23]. While the EA's tips are based on polymers that do not damage the canal wall, the ultrasonic system's irrigation tip is composed of metal alloys [24]. In straight canals, Blank-Goncalves et al. evaluated traditional irrigation against sonic and ultrasonic irrigation systems. Their results showed that compared to traditional needle irrigation in the canal, sonic and ultrasonic irrigation demonstrated superior smear layer removal; statistically, the difference between the two irrigation systems was insignificant [25].

To guarantee that the ultrasonic activation group's activation head would not harm the canal's inner walls, it was implanted 2 mm away from the WL without contacting the walls. According to the study's findings, sonic activation and PUI eliminated more smear layers than ordinary irrigation did. This finding is similar to the relevant literature and reinforces the benefits of ultrasonic and sonic activation methods [25]. PUI creates a continuous, time-dependent, unidirectional flow of fluid around a tiny vibrating object (Nyborg). Acoustic streaming cannot be produced by sonic activation, and its oscillation frequency is only 25% of PUIs. However, because it has a larger amplitude and its tip moves in three dimensions in an orbit, whereas the PUI oscillates transversely in a single plane, it is thought to have the same cleaning ability as the PUI [26].

XPF is composed of a tiny core with zero taper and an ISO 25 diameter. The file is in the martensite phase (20°) and is straight at room temperature. When placed into the canal at body temperature, it transits to the austenite phase (35°). Its apical section enlarges, like a spoon. According to reports, when the tip of the XPF file is squeezed, it may extend up to 6 mm in diameter, which is equivalent to 100 times the size of a file of the same size. This causes the file to be able to access locations that it is unable to access [13]. XPF is used with an endodontic motor, speed set to 800 rpm, and torque set to 1 N.cm. In line with other research, XPF had cleaner canal walls and reduced smear layer score in contrast with canal brush. The minimal mean score displayed by XPF is consistent with the research done by Mohanad Ghazi Azzawi. The XPF file's efficiency may be attributed to its tiny core size, which provides expansion capacity. The file has good adaptability and reaches every part of the canal wall [27]. In comparison with canal brush and traditional irrigation systems, at the apical level, XPFs result in lower scores for smear.

In comparison with the EA and passive ultrasonic groups, XPF group scored higher. The company that makes the XPF said that a synergistic effect on debridement was achieved by the apical preparation and vibration of this very flexible and sensitive file inside the continually provided fluid. But based on study's findings, XPF file did not maximize the EDTA's capability to eliminate debris and smear in vitro. This might be because metal tends to produce more debris and smear layers [28].

The canal brush, manually operated with a rotating motion, is made completely of polypropylene. Attaching it to a contra-angle handpiece that is operating at 600 rpm, however, increases its effectiveness. Set at 600 rpm, a single handpiece employs a polypropylene canal brush [22].

The better outcomes of the canal brush group than the control group are similar with earlier research conducted by Garip et al. and Narmatha et al. [29]. The friction created between the brush and the canal wall stops the solution from flowing inside, despite the brush's push and pull motion mechanically displacing the tissues. In this research, a slow speed handpiece was utilized for the canal brush in a circumferential and 1-2 mm up-and-down motion for 60 seconds. The wide apical preparation in the current investigation, with a size 30, 0.09 taper, allowed the canal brush (tip diameter 0.25 mm) to operate passively, allowing the irrigant to move freely. Additionally, this made the root canal system cleaner. However, the soft, flexible bristles of the canal brush are unable to identify the potential for mechanical influence because of the research design.

Protogerou et al. (2013) found no increase in cleanliness and no discernible differences between those using the canal brush and those who did not [30]. Despite being more extensive than the minimal preparation required for the medium-sized canal brush and in accordance with the company's recommendations, it appears that the smaller canal preparation (30/0.6) recommended in the study created an early bristle distortion and "squeezing" inside the proportionately narrow root canal when paired with the medium-sized canal brush. The brush may have created strong friction over the root canal walls as a result, reproducing the area that EDTA's demineralizing effect had previously cleaned. There is no tapering in the overall canal brush design. As a result, the instrumented root canal was forced by a parallel-form instrument to rotate into a tapering area. This indicates that the bristle deformation may be severe in the smallest diameter of the cone or apical one-third and that it may stop acting on the canal walls early.

## Conclusions

The EA removes the smear layer effectively as compared with PUI. XPF and root canal brush are the least efficient for smear layer eradication compared to EAs as well as PUI. So, to conclude, smear layer elimination mainly depends on the type of movement of irrigation tips and their speed during irrigation activation. The speed of the EA was 10,000 c/m, which was the maximum compared to the other activation devices. The tip of the EA has three-dimensional orbital movement, while the PUI oscillates transversely in one plane.
